# Mitochondrial Respiration-Dependent ANT2-UCP2 Interaction

**DOI:** 10.3389/fphys.2022.866590

**Published:** 2022-05-25

**Authors:** Tomas A. Schiffer, Liza Löf, Radiosa Gallini, Masood Kamali-Moghaddam, Mattias Carlström, Fredrik Palm

**Affiliations:** ^1^ Department of Physiology and Pharmacology, Karolinska Institutet, Solna, Sweden; ^2^ Department of Immunology, Genetics and Pathology, Science for Life Laboratory, Uppsala University, Uppsala, Sweden; ^3^ Department of Medical Cell Biology, Uppsala University, Uppsala, Sweden

**Keywords:** adenine nucleotide translocase-2, uncoupling protein-2, mitochondria, proximity ligation assay, protein interaction

## Abstract

Adenine nucleotide translocases (ANTs) and uncoupling proteins (UCPs) are known to facilitate proton leak across the inner mitochondrial membrane. However, it remains to be unravelled whether UCP2/3 contribute to significant amount of proton leak *in vivo*. Reports are indicative of UCP2 dependent proton-coupled efflux of C4 metabolites from the mitochondrial matrix. Previous studies have suggested that UCP2/3 knockdown (KD) contributes to increased ANT-dependent proton leak. Here we investigated the hypothesis that interaction exists between the UCP2 and ANT2 proteins, and that such interaction is regulated by the cellular metabolic demand. Protein-protein interaction was evaluated using reciprocal co-immunoprecipitation and *in situ* proximity ligation assay. KD of ANT2 and UCP2 was performed by siRNA in human embryonic kidney cells 293A (HEK293A) cells. Mitochondrial and cellular respiration was measured by high-resolution respirometry. ANT2-UCP2 interaction was demonstrated, and this was dependent on cellular metabolism. Inhibition of ATP synthase promoted ANT2-UCP2 interaction whereas high cellular respiration, induced by adding the mitochondrial uncoupler FCCP, prevented interaction. UCP2 KD contributed to increased carboxyatractyloside (CATR) sensitive proton leak, whereas ANT2 and UCP2 double KD reduced CATR sensitive proton leak, compared to UCP2 KD. Furthermore, proton leak was reduced in double KD compared to UCP2 KD. In conclusion, our results show that there is an interaction between ANT2-UCP2, which appears to be dynamically regulated by mitochondrial respiratory activity. This may have implications in the regulation of mitochondrial efficiency or cellular substrate utilization as increased activity of UCP2 may promote a switch from glucose to fatty acid metabolism.

## Introduction

Adenine nucleotide translocases (ANTs) are the most abundant proteins in mitochondria and account for up to 1% of the total mitochondrial protein content (Brand et al., 2005). Functional forms of ANTs are present in monomers and dimers (Ardalan et al., 2021b; Nicholls, 2021). The main function of the ANT isoforms (*i.e.,* ANT 1–4) is to catalyse the 1:1 electrogenic exchange of newly synthesized ATP for cytosolic localized ADP across the inner mitochondrial membrane. The charge imbalance between ATP and ADP drives the efflux of ATP and influx of ADP (Klingenberg, 2008). ANT isoforms are categorized by their tissue-specific transcriptional expression; ANT1 is the predominant isoform in heart and skeletal muscle (Liu and Chen, 2013), ANT2 is mostly found in kidney, spleen, liver, fibroblasts and lymphocytes (Stepien et al., 1992; Doerner et al., 1997), whereas ANT3 is ubiquitously expressed in most tissues (Stepien et al., 1992). ANT4 is exclusively expressed in the testis, liver and brain (Dolce et al., 2005; Dupont and Stepien, 2011).

Besides their crucial role in oxidative phosphorylation, ANTs have been identified as a part of the permeability transition pore (Bernardi et al., 2015; Halestrap and Richardson, 2015) and are involved in the regulation of the Ca^2+^-dependent sensitivity of pore opening (Halestrap, 2009). Ca^2+^ sensitivity is decreased upon trapping ANT in m-conformation with bongkrekik acid, whereas locking it in c-conformation with carboxyatractyloside (CATR) increases Ca^2+^ sensitivity (Hunter and Haworth, 1979; Halestrap et al., 1997; Haworth and Hunter, 2000). ANT has been shown to interact with various subunits of the electron transport chain complexes (Lu et al., 2017; Chorev et al., 2018). Ya-Wen Lu and colleagues (Lu et al., 2017) showed that human ANT assembled in a range of complexes from 67 to >669 kDa, and COX IV was also shown to co-immunoprecipitate with ANT2. In addition, numerous mitochondrial carriers have been shown to assemble with ANT1 and ANT2 (Lu et al., 2017). Interestingly, knockdown (KD) of ANT2 improved coupling efficiency and reduced proton leak dependent respiration, whereas overexpressing any of the ANT isoforms increased basal respiration (Lu et al., 2017).

ANT has an intrinsic property of mediating proton leak across the mitochondrial inner membrane, contributing to basal proton leak, which has been coupled to basal metabolic rate (Brand et al., 2005). ANT-mediated proton leak is known to be induced by fatty acids (Andreyev et al., 1989) and oxidative stress-modified lipid peroxidation products such as 4-hydroxy-2-nonenal (HNE) (Echtay et al., 2003). Recently, it has been shown that ANT1 and ANT2 could participate in *N*-acyl amino acid–associated uncoupled respiration (Long et al., 2016). Overexpressing ANT3 and ANT2 generates increased ANT-specific transport and increased basal and maximal respiration, respectively (Lu et al., 2017), indicative of isoform-specific properties. A recent study suggested that ANT serves as a master regulator of mitochondrial energy output, contributing to a delicate balance between ATP production and thermogenesis, where the proton conductance is negatively regulated by ADP/ATP exchange (Bertholet et al., 2019). Furthermore, cold-induced expression of ANT in chicken (Toyomizu et al., 2002) and rat skeletal muscle (Boss et al., 1997) suggests involvement in thermoregulation (Toyomizu et al., 2002). Similarly, with ANTs, uncoupling proteins (UCPs) are known to contribute to proton leak across the mitochondrial inner membrane and thereby induce a mild reduction in membrane potential. UCP1 is responsible for the adaptive non-shivering thermogenesis (Nicholls et al., 1978; Matthias et al., 2000; Golozoubova et al., 2001), whereas proton leak through UCP2 and UCP3 has been suggested to be under the regulation of superoxide and HNE (Brand et al., 2004; Malingriaux et al., 2013). In addition, deglutathionylation during oxidative stress may activate proton leak through UCP2 which is thought to be implicated in reducing superoxide production (Mailloux and Harper, 2011).

In a skeletal muscle UCP3 knockout model, mitochondria were more coupled and this was associated with increased production of reactive oxygen species (ROS) (Vidal-Puig et al., 2000). Similarly to ANT, UCP3 expression was shown to be cold-induced in chickens (Toyomizu et al., 2002). In addition, 3,4-methylenedioxymethamphetamine (MDMA)-induced thermogenic response is almost completely abolished in mice lacking UCP3 (Mills et al., 2003).

However, whether UCP2/3 contribute to physiologically relevant levels of proton leak *in vivo* is still under debate. No, difference in proton leak in skeletal muscle mitoplasts was observed in UCP2/UCP3 KO mice compared to WT and the proton leak was guanosine diphosphate (GDP) insensitive (Bertholet et al., 2019). Many studies have shown that UCP2/3 are required to prevent oxidative stress, however, the underlying mechanisms remain elusive and are still a subject of debate. Controversies surrounding ROS- and HNE-dependent activation of UCP2/3-mediated proton leak has been addressed in several reviews (Cannon et al., 2006; Nicholls 2021). Recently, Ardalan and colleagues proposed the formation of tetrameric (dimer of stable dimers) UCP2 in which each monomer within the dimers are in the same transport mode (cytoplasmic or matrix states) (Ardalan et al., 2021a; Ardalan et al., 2021b). Matrix and cytoplasmic salt-bridge networks seem to be key controllers of the different states of UCP2 (Ardalan et al., 2021a).

Vozza and colleagues showed that UCP2 is utilizing efflux of C4 citric acid cycle metabolites malate, oxaloacetate, aspartate and malonate in exchange for inorganic phosphate and H^+^ (Vozza et al., 2014). They concluded that UCP2 works as a “break” in glucose/pyruvate metabolism by reducing oxaloacetate as the availability regulates the oxidation of pyruvate-derived acetyl-CoA (Bouillaud, 2009; Vozza et al., 2014). The results are in line with the observed fasting-induced UCP2 expression (Boss et al., 1997), which thereby induces a switch from glucose towards fatty acid metabolism (Pecqueur et al., 2008).

Interestingly, UCP2 or UCP3 deficiency seems to contribute to increased proton leak through ANT (Bevilacqua et al., 2010; Friederich-Persson et al., 2012) suggesting an existing crosstalk between ANT:s and UCP:s. Components of the respiratory system are known to form super-complexes (Acin-Perez et al., 2008; Moreno-Lastres et al., 2012; Lu et al., 2017; Guo et al., 2018), but it has not been investigated whether any interaction(s) exist between ANT:s and UCP:s. Brand and colleagues (Parker et al., 2008) observed that UCP3 dependent proton leak was abolished upon pre-inhibition of ANT with CATR or bongkrekik acid (BKA). They speculated that there might exist a direct interaction between ANT and UCP or via other proteins (Parker et al., 2008). In this study we aimed to establish whether an interaction exists between UCP2 and ANT2 and if such interaction is dependent on cellular metabolic demands as it may contribute to the regulation of substrate utilization during alterations in cellular metabolism.

## Material and Methods

### Animals

All animal procedures were evaluated and approved by the regional ethical committee in Uppsala and Stockholm, and were performed in accordance with the National Institutes of Health Guide for the Care and Use of Laboratory Animals. Male Sprague-Dawley rats (Charles River, Sulzfeldt, Germany), 12–16 weeks old, and were housed in a light-dark, temperature and humidity-controlled environment, and given *ad libitum* access to standard rodent chow and tap water until euthanization by decapitation followed by immediate excision of the kidneys.

### Mitochondrial Isolation and Leak Respiration

Mitochondria from the rat renal cortex were isolated by differential centrifugation, as described elsewhere (Schiffer et al., 2018). Mitochondrial respiration was measured using high-resolution respirometry (Oroboros 2K, Innsbruck, Austria). Arachidonic acid (2 μM) was used to initiate mitochondrial proton leak.

The GDP and ANT inhibitor sensitive leak respiration was defined as the change in proton leak-dependent respiration, when adding the selective ANT inhibitors carboxyatractyloside (CATR; 5 μM), bongkrekik acid (BKA; 5 μM) and GDP (2 mM) in the presence of pyruvate (5 mM), malate (2 mM) and oligomycin (2.5 μM). Mitochondrial integrity was confirmed by measuring respiratory control ratio (RCR) defined as maximal complex I mediated oxidative phosphorylation capacity (pyruvate (5 mM), malate (2 mM) and ADP (2.5 mM)) divided by leak state without adenylates. Mitochondrial respiration was evaluated in respiration medium containing EGTA 0.5 mM, MgCl_2_*6 H_2_O 3 mM, K-lactobionate 60 mM, taurine 20 mM, KH_2_P0_4_ 10 mM, HEPES 20 mM, Sucrose 110 mM, pH 7.1. Respiration was normalized to mitochondrial protein.

### Co-Immunoprecipitation of ANT2 and UCP2

We sought to evaluate UCP2-ANT2 interaction by co-immunoprecipitation and immunoblot analysis of endogenous proteins. The kidney cortex was collected, as described above, and immediately after dissection, samples were snap-frozen in liquid nitrogen. Samples were stored at −80°C until processed for analysis. A mortar was placed in liquid nitrogen and samples were cryogrinded to a fine powder with a pestle. Samples were lysed in lysis buffer provided by the commercial kit (Dynabeads™, CO-immunoprecipitation kit; Invitrogen, Carlsbad, California, United States) adjusted to 100 mM NaCl. ANT2 and UCP2 were immunocaptured from rat kidney cortex by using monoclonal antibodies against UCP2 (#89326; Cell Signaling, Danvers, Massachusetts, United States) and ANT2 (#14671; Cell Signaling) diluted in Phosphate Buffered Saline (PBS). Antibodies were cross-linked to magnetic beads (Dynabeads™, Co-immunoprecipitation kit; Invitrogen) following the manufacturer’s instructions. Lysates were incubated with magnetic beads at room temperature for 35 min with rotation before the purification steps were initiated. The final sample was diluted with Laemmi buffer (4x Laemmli sample buffer (Bio-Rad Laboratories, Hercules, California, United States), 5% beta-mercaptoethanol) and heated at 95°C for 5 min. Immune complexes were separated by SDS-PAGE using precast gradient gels (Criterion, TGX, 4–20% (Bio-Rad). Proteins were transferred to a polyvinylidene difluoride membrane. Membranes were blocked with TBS, containing 0.2% Tween-20 and 5% bovine serum albumin (BSA). Primary antibodies targeting UCP2 (#89326; Cell Signaling) and ANT2 (#14671; Cell Signaling) were diluted 1:1000 in Tris-buffered saline, 0.1% Tween 20, 5% BSA. Stripping buffer was used between immunoblotting following the manufacturer’s instructions (Restore western blot, stripping buffer; Thermo Fisher Scientific, Waltham, Massachusetts, USA).

### Cell Culture

HEK293 cells were grown at 37°C and 5% CO_2_ in DMEM supplemented with 10% fetal bovine serum (FBS), 50 units/ml penicillin, 50 μg/ml streptomycin and 1 mM l-glutamine, 0.1 mM MEM Non-Essential Amino Acids (NEAA) (all from Thermo Fisher Scientific). The cells were tested for mycoplasma using Mycoplasma Detection Kit-Quick Test (Biotool, Jupiter, Florida, United States).

### Native PAGE

HEK293 cells were harvested and lysed in RIPA buffer. Native PAGE was performed to confirm the binding of antibodies to the native target protein used during *in situ* PLA. Electrophoresis was performed at 90V using precast gels on ice in a cold room (Mini-PROTEAN TGX 4–15%; Bio-Rad). The running buffer contained: Tris-base, 30g/L and glycine 144 g/L. Transfer of proteins to PVDF membrane was performed at 30V for 16h on ice in a cold room using Bjerrum transfer buffer containing: 58.2 g/L Tris-base and 29.3 g/L glycine and 0.04% SDS (pH 9.2). Membranes were washed for 20 min with Bjerrum transfer buffer without SDS before blocked with 5% milk in TBST (0.1% Tween 20). Membranes were incubated with primary antibodies overnight with either mouse anti-UPC2 (1:1000, orb333895; Biorbyt) or rabbit anti-ANT2 (1:1000, #14671; Cell Signaling) primary antibodies. After washing in TBST, membranes were incubated with secondary HRP-linked anti-rabbit or anti-mouse antibodies (1:10000, #7074S, #7076S; Cell Signaling). Blots were developed using SuperSignal West Femto Maximum Sensitivity Substrate (Thermo Fisher Scientific) and intensities were quantified using densitometry (ChemiDoc MP Imaging System; Bio-Rad).

### *In situ* Proximity Ligation Assay

Human embryonic kidney cells 293 (HEK 293) cells were seeded at a density of approximately 120,000 cells/cm^2^ and grown for 1–2 days in 8-well Nunc Lab-Tek II CC2 Chamber Slides (Thermo Fisher Scientific) until confluency reached 70–80%.

The cells were subsequently incubated in Dulbecco's Modified Eagle's Medium (DMEM) at 37°C with or without 2.5 µM oligomycin or 0.5 µM FCCP for 2 min, respectively. The cells were washed twice in 1x PBS before fixation with pre-warmed 3.7% formaldehyde (F8775; Sigma-Aldrich, Saint Louis, Missouri, United States) with 5% sucrose (84097; Sigma-Aldrich) in 1x PBS (pH 7.4) for 20 min at 37°C. After rinsing once with 1x PBS, permeabilization was carried out with ice-cold acetone for 10 min at -20°C (00570; Honeywell Fluka, Munich, Germany,) and rinsed again with 1x PBS.

*In situ* PLA was performed using a standard protocol at PLA and Single Cell Proteomics Facility SciLifeLab, Uppsala University, as previously described (Soderberg et al., 2006). To reduce the likelihood of unspecific binding of PLA probes, the HEK 293 cells were blocked at 37°C for 1h in Duolink blocking solution (DUO92003, MilliporeSigma, Burlington, Massachusetts, United States). Cells were incubated at 4°C overnight with mouse anti-UPC2 (1:250, orb333895; Biorbyt, Cambridge, United Kingdom) and rabbit anti-ANT2 (1:750, #14671; Cell Signaling) primary antibodies, washed for 5 min for 3 times with TBST, followed by 1h incubation at 37°C with donkey anti-mouse and donkey anti-rabbit (715–005–151 and 715–005–152; Jackson ImmunoResearch, West Grove, Pennsylvania, United States) conjugated to the priming and non-priming proximity probe oligonucleotides, as described by Söderberg *et al.* (Soderberg et al., 2006). Cells were washed in Tris-buffered saline, 0.1% Tween20 (TBST) and incubated for 30 min at 37°C in a ligation/hybridization solution. Following another wash in TBST, the PLA signals were amplified for 90 min at 37°C. The detection oligonucleotides were labelled with Cy3 fluorophore. The ligation/hybridization, amplification solution and protocol, as well as the sequences of the connector and detection oligonucleotides, are described by Söderberg *et al.* (22). Nuclei were visualized by Hoechst incubation. Cell slides were mounted for microscopy analysis using SlowFade Gold Antifade Reagent (S36936; Thermo Fisher Scientific). A single primary antibody with PLA probes and PLA probes without primary antibodies were routinely included as negative controls. PLA signals from samples were subtracted with background PLA signals from the negative controls before running statistical analysis.

### Knockdown of UCP2 and ANT2

HEK293A cells were grown under similar conditions. At 60–80% confluence, cells were transfected with siRNA targeting ANT2 and UCP2 (Thermofisher, Waltham, Massachusetts, U nited States). Using Lipofectamine RNAiMAX as transfection reagent following the manufacturer’s instructions (Thermo Fisher Scientific). UCP2 and ANT2 knockdown (KD) were confirmed by qPCR. Primer sequence for UCP2 forward: AAG​GTC​CGA​TTC​CAA​GCT​CA, reverse: TCA​GCA​CAG​TTG​ACA​ATG​GC. ANT2 forward: GGC​TTT​AAC​GTG​TCT​GTG​CA, reverse: ATA​GGA​AGT​CAA​CCC​GGC​AA.

### Cell Respiration

Basal cell respiration was determined by high-resolution respirometry (Oroboros 2K). Cells were permeabilized with digitonin (∼4 μM). Rotenone (0.5 μM), succinate (10 μM) and oligomycin (10 nM) were added to measure leak respiration. CATR (5 μM) was added to determine CATR sensitive respiration. Respiration was normalized to cell number. Viability was determined with trypan blue.

### Statistics

Paired student’s t-test was used when comparing differences in mitochondria isolated from the same animal. Unpaired Student´s t-test was performed when analysing differences in isolated mitochondria between animals assuming Gaussian distribution. A one-way ANOVA followed by Tukey’s multiple comparisons test was used when comparing more than two groups. Non-parametric Mann-Whitney test was performed when evaluating partial differences between inhibitor specific influence on UCP and ANT inhibition. *p* < 0.05 was considered statistically significant. * denotes *p* ≤ 0.05, **p ≤ 0.01, and ***p ≤ 0.001 respectively, and non-significant differences are indicated as n.s.

## Results

### Respirometry

**FIGURE 1:**
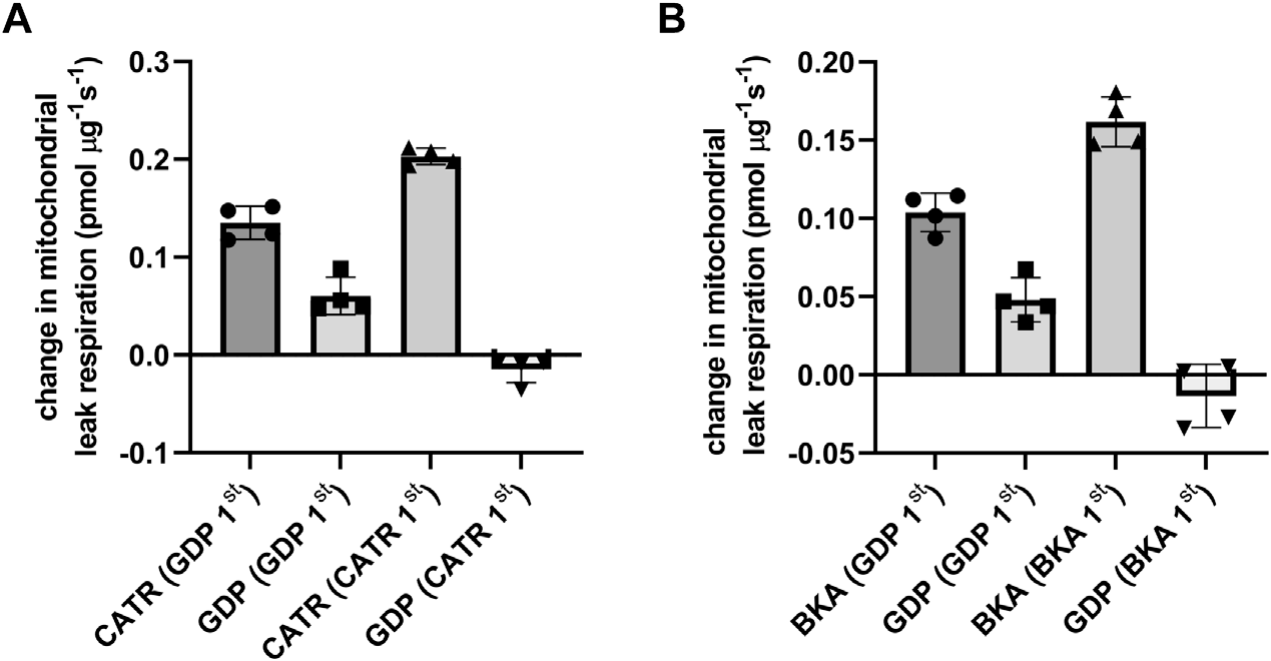
Inhibitor order specific ANT- and UCP-dependent leak respiration were measured in isolated rat kidney mitochondria and defined as the change in leak respiration by adding the ANT specific inhibitor CATR locking ANT into c-conformation and the UCP inhibitor GDP during mitochondrial leak state in presence of pyruvate, malate and oligomycin. Before ANT and UCP inhibition, leak mediated respiration was induced by arachidonic acid. The order of inhibition is stated underneath the x-axis. **(A)** Inhibitor order specific ANT and UCP dependent leak respiration using CATR and GDP. **(B)** In a separate experiment with similar conditions, ANT inhibitor BKA (locking ANT in m-conformation) was used.

The respiratory control ratio confirmed mitochondria with high integrity after the isolation process (9,7± 0.4). Inhibition order specific effects on ANT and GDP-sensitive mediated mitochondrial proton leak was evaluated using the ANT inhibitors CATR (locking ANT in c-conformation) or BKA (locking ANT in m-conformation) together with GDP as UCP inhibitor. UCP2 inhibition prior to CATR (Figure 1A) or BKA (Figure 1B) revealed an approximately 30/70 contribution of GDP-sensitive and ANT specific proton leak. On the other hand, inhibiting ANT with either CATR or BKA abolished further GDP-sensitive proton leak (Figure 1A, Figure 1B), showing similarities between UCP2 and UCP3 previously reported by Parker and colleagues (Parker et al., 2008). Total leak respiration did not differ between animals receiving CATR (255 ± 25 pmol mg^−1^ s^−1^) or BKA (230 ± 8 pmol mg^−1^s^−1^).

### Co-Immunoprecipitation of ANT2 and UCP2

**FIGURE 2:**
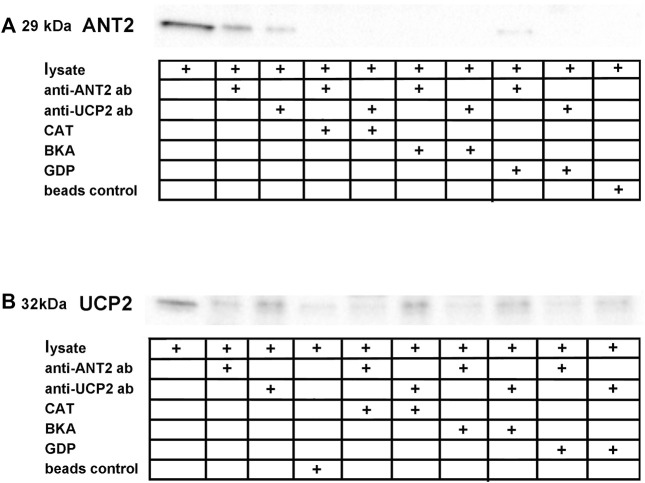
Reciprocal co-immunoprecipitation was performed on lysate extracted from rat kidney cortex using magnetic beads coupled to anti-ANT2 and anti-UCP2 antibodies. **(A)**, ANT2 co-purified with UCP2 when using beads coupled to anti-UCP2 antibodies. ANT and UCP inhibitors interfered with the ANT2-UCP2 interaction. **(B)**, Similarly, UCP2 co-purified with ANT2 when using beads coupled to anti-ANT2 antibodies. The lane representing beads control revealed binding between the magnetic beads and UCP2, although to a smaller extent than in the lane representing the ANT2. The same inhibitor specific interaction interference was observed, as the band densities did not exceed the apparent unspecific binding between the beads and UCP2.

Reciprocal co-immunoprecipitation was performed on rat kidney cortex tissue lysates using ANT2 and UCP2 coupled to magnetic beads and the samples were immunoblotted with either anti-ANT2- or anti-UCP2 antibodies. The band representing ANT2 (Figure 2A) was co-immunoprecipitated with UCP2 using the anti-UCP2 antibody. Bands were absent when lysates were incubated with CATR or BKA prior to the co-immunoprecipitation protocol. Incubating samples with GDP prevented the protein association. Similarly, the weaker band representing UCP2 (Figure 2B) was co-immunoprecipitated with ANT2 when using the anti-ANT2 antibody. The signal in the bands representing UCP2 when incubating samples with CATR and BKA and GDP did not exceed the background signal representing the non-selective binding of UCP2 to the magnetic beads in the lane with only beads as control.

### *In situ* Proximity Ligation Assay

**FIGURE 3:**
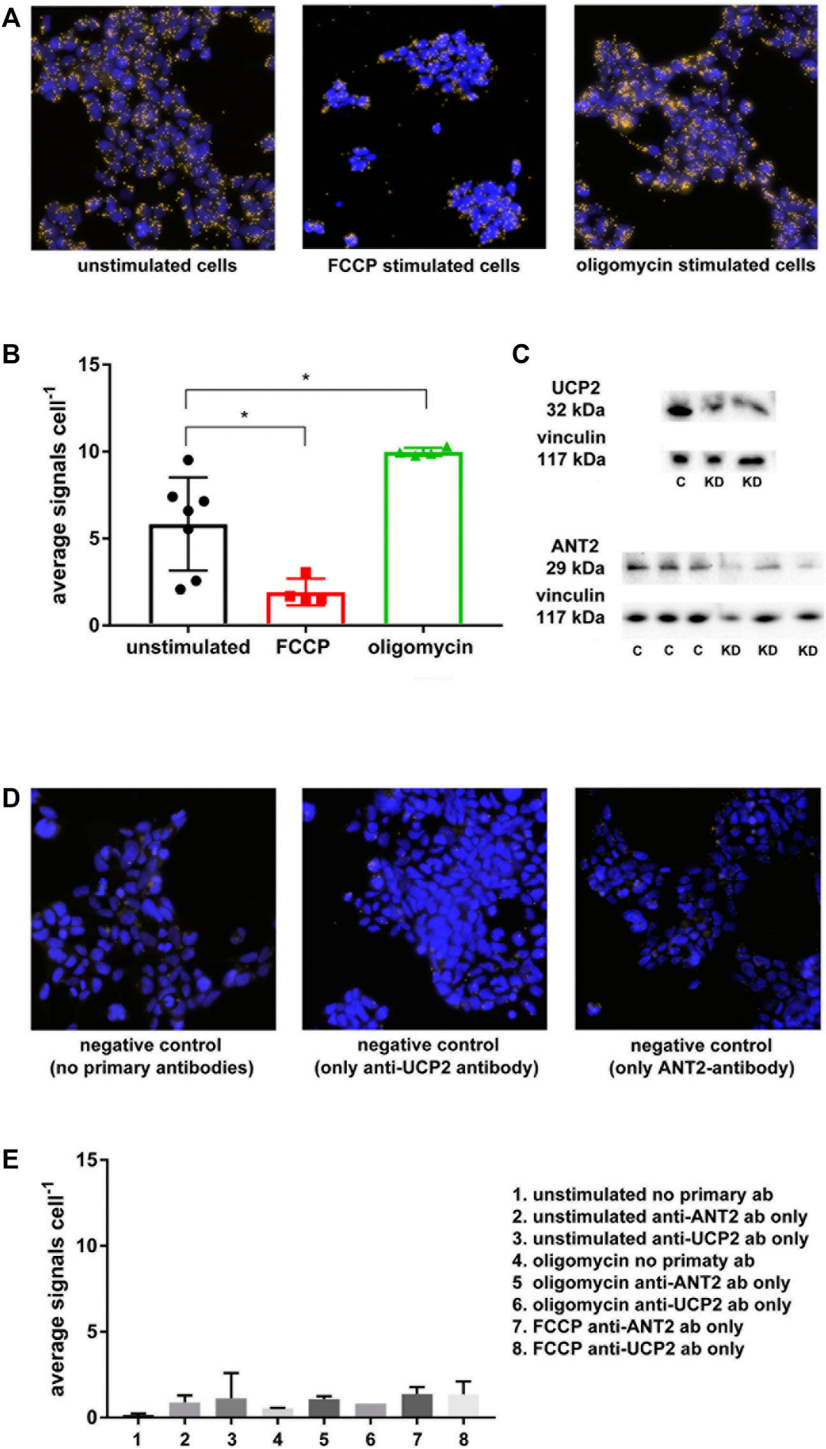
*In situ* PLA was performed on HEK293 cells using anti-ANT2 and anti-UCP2 antibodies. Yellow dots represent a within approximately 40 nm co-localization of UCP2 and ANT2. **(A)** *Unstimulated cells* represent cells at basal condition, whereas *FCCP stimulated cells* represent artificially induced mitochondrial respiration and lowering of membrane potential via mitochondrial uncoupling. *Oligomycin stimulated cells* represent the lowest possible respiration together with the highest possible membrane potential via ATP-synthase inhibition with oligomycin. **(B)**, Graph displaying the extent of ANT2-UCP2 interaction dependent on cellular respiration explained in **(A)**. **(C)** Native PAGE was performed to confirm binding of the anti-UCP2 and anti-ANT2 to the native proteins. **(D)** Negative controls using only secondary antibodies or secondary antibodies in presence of either primary anti-ANT2 or anti-UCP2 antibodies. **(E)** Graph representing negative controls displayed as the average number of PLA signals per cell.

The *in situ* PLA confirmed co-localization between ANT2 and UCP2 at basal cellular conditions with a reduction of the signal upon FCCP stimulation and an increased signal after treating cells with oligomycin (Figures 3A,B). A significantly lower number of PLA signals per cell were observed during FCCP-mediated mitochondrial uncoupling compared to those of unstimulated cells, which was indicative of dissociation between UCP2 and ANT2 at higher respiration and lower membrane potential (Figures 3A,B). Furthermore, artificially minimizing mitochondrial respiration *via* ATP-synthase inhibition with oligomycin leading to maximized membrane potential, significantly increased the number of PLA signals per cell compared to unstimulated cells (Figures 3A,B). This is indicative of an association between UCP2 and ANT2 at low respiration and high membrane potential. Native PAGE confirmed binding of primary antibodies to the native target proteins UCP2 and ANT2 (Figure 3C). Negative controls with secondary antibodies together with either anti-ANT2 or anti-UCP2 primary antibodies assured the absence of interaction between antibodies (Figures 3D,E). Various negative controls with oligomycin and FCCP present ensured that substances did not influence the signals (Figures 3D,E).

### Knockdown of UCP2 and ANT2 in HEK293A Cells

**FIGURE 4:**
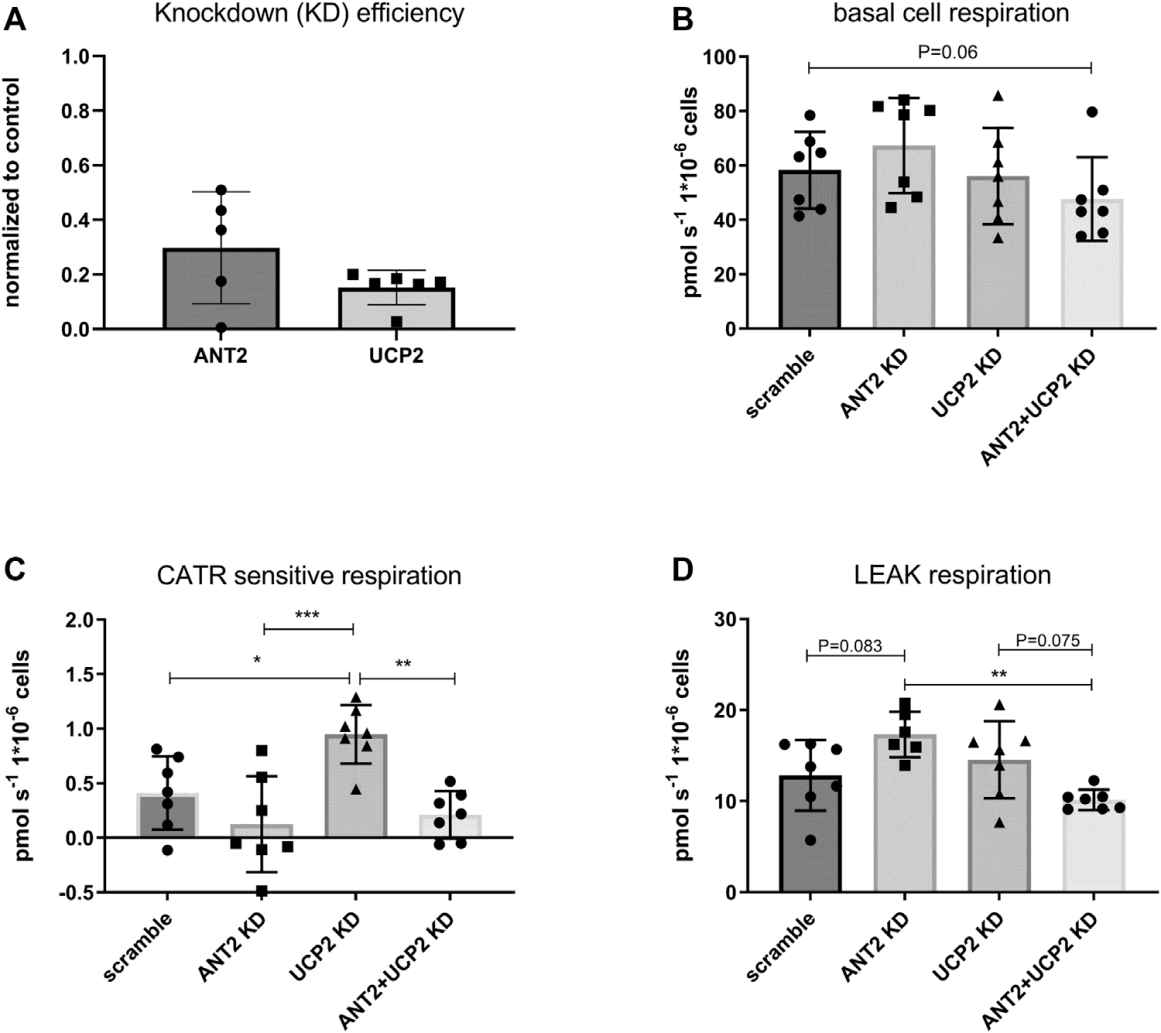
ANT2 and UCP2 were knocked down (KD) in HEK293A cells using siRNA. The efficiency of KD by siRNA was evaluated by rtqPCR **(A)**. Cells were harvested and diluted in respiration medium. Basal cell respiration was measured by high-resolution respirometry. Respiration was normalized to cell number. Basal cell respiration was measured after ANT2 and UCP2 KD. **(B)**. CATR sensitive respiration was determined in permeabilized cells and defined as the change in respiration after the addition of CATR during leak respiration. **(C)**. Leak respiration was measured in permeabilized cells (digitonin) in presence of complex II substrates (succinate) rotenone and oligomycin **(D)**.

Real-time qPCR confirmed approximately 70% efficiency of ANT2 KD and 85% efficiency of UCP2 KD (Figure 4A). Basal respiration trended to decrease in the double KD whereas it was unchanged in the UCP2 KD or ANT2 KD group (Figure 4B). UCP2 KD contributed to significantly increased CATR sensitive respiration (Figure 4C). Leak respiration trended to increase upon ANT2 KD and was significantly higher than the double KD (Figure 4D).

## Discussion

The main finding of this study is the observed interaction between ANT2 and UCP2 and that this interaction is dependent on cellular metabolism. ANT is changing its conformation between facing the cytosolic (c-state) or the matrix side (m-state) (Yamamoto et al., 2015), where CATR and BKA lock ANT in c-state and m-state, respectively (Pebay-Peyroula et al., 2003; Yamamoto et al., 2015). At low cellular metabolism, *i.e.,* high membrane potential, ANT favours m-state conformation, where it has previously been shown to be more prone to promote proton leak *via* HNE activation (Azzu et al., 2008; Bertholet et al., 2019). In our experimental setup, we measured ANT sensitive respiration at leak state during ATP-synthase inhibition which potentiates the membrane potential, where ANT favours m-state. GDP-sensitive inhibition was abolished after prior ANT inhibition by adding CATR or BKA (Figures 1A,B) indicating that UCP2 dependent proton leak is inhibited upon ANT inhibition, suggesting interaction between proteins. Previous reports showed similar effects for UCP3 (Parker et al., 2008; Komelina and Amerkhanov, 2010) However, GDP is shown to be an unspecific inhibitor of UCPs and partly inhibits ANTs as well (Komelina and Amerkhanov, 2010).

The reciprocal co-purification of ANT2 and UCP2 supported an existing protein-protein interaction between ANT2 and UCP2 (Figures 2A,B). The absence of ANT2 in the samples treated with either CATR or BKA is intriguing, suggesting that the inhibitors prevented the binding between the primary antibody and ANT in the native state or that the extreme transition states c- or m- prevented interaction. Firm conclusions regarding ANT2 conformation-specific dependent ANT2-UCP2 interaction was not possible to conduct in this setup. The beads control indicated unspecific binding between beads and UCP2, which made the interpretation of the reciprocal approach somewhat questionable. However, in support of the co-purification experiments, *in situ* PLA confirmed co-localization of ANT2 and UCP2 (Figures 3A,B) (Bagchi et al., 2015). ANT is known to form super-complexes with other components in the respiratory chain (Lu et al., 2017), and has also been shown to interact with voltage-dependent anion channel (VDAC) (Allouche et al., 2012). The *in situ* PLA establishes co-localization but it is not possible to rule out ANT2-UCP2 interaction via other protein members of an existing super-complex. Importantly, the ANT2-UCP2 co-localization was highly dependent on cellular metabolism, where boosting mitochondrial respiration using the mitochondrial uncoupler FCCP promoted dissociation between ANT2 and UCP2, whereas minimizing respiration by adding oligomycin promoted co-localization.

Although not supported by the co-purification experiments and therefore speculative, the ANT2-UCP2 interaction may be ANT2 conformation-dependent as ANT2 predominantly is in c-state during high mitochondrial respiration (high ADP/ATP exchange) and predominantly in m-state during low respiration and high membrane potential (Azzu et al., 2008). Indications of structural similarities between ANT and UCP2 have previously been reported (Berardi et al., 2011). However, a recent molecular dynamics analysis indicated that there are striking differences in the structure between these proteins (Zoonens et al., 2013). Monomeric, dimeric (Klingenberg and Appel, 1989; Jekabsons et al., 2003) and tetrameric forms (dimer of dimers) of functional UCP2 have been reported (Hoang et al., 2015). Ardalan and colleagues showed that the tetrameric form of UCP2 was capable of transporting protons in lipid layers where the two sets of dimers of the tetramer were present in different conformations, *i.e.*, two being in the m-state and the other two in c-state (Ardalan et al., 2021b). The work by Ardalan, showed conformational changes of UCP2 upon oligomerization. There is a possibility that interaction between ANT and UCP2 may contribute to additional conformational changes in both directions which may have implications on function and proton conductance via these proteins. In addition, the presence of ATP known to have inhibitory effects on UCP:s may play a role in interaction between proteins. Concerns have been raised regarding detergent induced structural perturbations in some proteins which can make the functional interpretations after reconstitution in lipid layers questionable (Zhou and Cross, 2013). To date, UCP1 is the only accepted “true uncoupler” reviewed in, (Nicholls, 2021). The troubled history surrounding UCP2/3 in the prevention of oxidative stress and the transport of protons was recently highlighted (Nicholls, 2021).

Knocking down either ANT2 or UCP2 in HEK293A cells had no significant effect on basal cell respiration although trending to be attenuated in the double KD (Figure 4B). Unexpectedly, the CATR sensitive respiration was not significantly changed in ANT2 KD cells compared to controls. The explanation may be that the protein levels of ANT2 was not efficiently knocked down as indicated by the variability of the rtqPCR (Figure 4A) and the native PAGE results which indicated approximately 50% KD (Figure 3C). Nevertheless, although not reaching significance, ANT2 KD seemed to contribute to increased leak respiration which is likely not explained by increased leak *via* other ANT isoforms as the double KD presented a significantly lower proton leak dependent respiration compared to the ANT2 KD alone (Figure 4D). Furthermore, the CATR sensitive respiration in the double KD was not different from the ANT2 KD (Figure 4C). The results suggest that the proton conductance *via* UCP2 increased because of ANT2 KD which contradicting the recent findings by Vozza and others that UCP2 does not contribute to a significant proton leak (Vozza et al., 2014). As previously reported, the CATR sensitive respiration increased upon UCP2 KD (Figure 4C) (Bevilacqua et al., 2010; Friederich-Persson et al., 2012). The explanation may be a superoxide-dependent activation of ANT mediated leak respiration because of increased membrane potential in the absence of UCPs (Echtay et al., 2003) and vice versa. It may also depend on the attenuation of interaction between ANT2/UCP2 in the KD which would then suggest that the interaction between proteins may work as a break in the proton leak via each protein. However, the PLA assay showed an induced co-localization of ANT2-UCP2 during oligomycin treatment corresponding to a low ADP/ATP exchange previously shown to contribute to maximized proton leak *via* ANT (Vozza et al., 2014) arguing against the previous reasoning.

Vozza and colleagues showed UCP2 likely to be a transporter of C4 metabolites, rather than a proton uncoupler (Vozza et al., 2014) although the transport of these metabolites seemed to be facilitated by H^+^. Bertholet and colleagues argued that UCP2/3 does not contribute to proton leak as currents could not be detected through these proteins (Bertholet et al., 2019). However, it is unclear whether ANT was present in c-conformation during their experimental conditions.

As the proton conductance or transport of C4 metabolites via UCP2 may be dependent on ANT2 interaction, it would be of interest to investigate whether interaction may alter UCP2 activity. Moreover, ANT is subjected to post-translational modifications such as phosphorylation (Feng et al., 2008; Cesaro and Salvi, 2010; Zhao et al., 2011), acetylation (Mielke et al., 2014), S-nitrosylation (Chang et al., 2014), glutathionylation (Queiroga et al., 2010), and carbonylation (Yan and Sohal, 1998). The effect of such modifications on ANT dependent proton leak and the influence on the ANT-UCP interaction is currently unknown and warrants further investigations.

It is not possible to distinguish between specific UCP and ANT-isoforms when using the inhibitors available. Although ANT2 and UCP2 are the far most abundant isoforms in the renal cortex (Stepien et al., 1992; Doerner et al., 1997; Friederich et al., 2009), the respiratory results may also involve interactions between other isoforms present in the samples. In our study, co-immunoprecipitation and *in situ* PLA were performed on UCP2 and ANT2 isoforms only. The absence of specific UCP inhibitors is a limitation of the study, as the promiscuity of GDP that is commonly used as a UCP inhibitor also contributes to inhibition of proton leak *via* ANTs (Khailova et al., 2006; Parker et al., 2008; Komelina and Amerkhanov, 2010).

In conclusion, this study demonstrates an existing interplay between ANT2 and UCP2, which is dependent on cellular metabolism. This interaction may influence ANT2/UCP2 dependent proton leak. As UCP2 seems to be involved in the efflux of C4 metabolites in the mitochondria, this interaction may also have implications in the regulation of cellular substrate utilization as increased activity of UCP2 can promote a switch from glucose to fatty acid metabolism. ANT dependent increased UCP2 activity, at low cellular ATP demand, may contribute to a shift towards fatty acid oxidation, whereas a lower activity at higher cellular ATP demand may contribute to a switch towards glucose utilization. Future studies will reveal the functional significance of this interaction and if one may potentially target this pharmacologically to modulate balance between glucose and fatty acid metabolism.

## Data Availability

The original contributions presented in the study are included in the article/Supplementary Materials, further inquiries can be directed to the corresponding author.

## References

[B1] Acín-Pérez R. Fernández-Silva P. Peleato M. L. Pérez-Martos A. Enriquez J. A. (2008). Respiratory Active Mitochondrial Supercomplexes. Mol. Cell. 32 (4), 529–539. 10.1016/j.molcel.2008.10.021 19026783

[B2] Allouche M. Pertuiset C. Robert J.-L. Martel C. Veneziano R. Henry C. (2012). ANT-VDAC1 Interaction Is Direct and Depends on ANT Isoform Conformation *In Vitro* . Biochem. Biophysical Res. Commun. 429 (1-2), 12–17. 10.1016/j.bbrc.2012.10.108 23131554

[B3] Andreyev A. Y. Bondareva T. O. DedukhovaMokhova V. I. E. N. Mokhova E. N. SkulachevVolkov V. P. N. I. Tsofina L. M. (1989). The ATP/ADP-antiporter Is Involved in the Uncoupling Effect of Fatty Acids on Mitochondria. Eur. J. Biochem. 182 (3), 585–592. 10.1111/j.1432-1033.1989.tb14867.x 2546761

[B4] Ardalan A. Sowlati-Hashjin S. Oduwoye H. Uwumarenogie S. O. Karttunen M. Smith M. D. (2021a). Biphasic Proton Transport Mechanism for Uncoupling Proteins. J. Phys. Chem. B 125 (32), 9130–9144. 10.1021/acs.jpcb.1c04766 34365794

[B5] Ardalan A. Sowlati-Hashjin S. Uwumarenogie S. O. Fish M. Mitchell J. Karttunen M. (2021b). Functional Oligomeric Forms of Uncoupling Protein 2: Strong Evidence for Asymmetry in Protein and Lipid Bilayer Systems. J. Phys. Chem. B 125 (1), 169–183. 10.1021/acs.jpcb.0c09422 33373220

[B6] Azzu V. Parker N. Brand M. D. (2008). High Membrane Potential Promotes Alkenal-Induced Mitochondrial Uncoupling and Influences Adenine Nucleotide Translocase Conformation. Biochem. J. 413 (2), 323–332. 10.1042/BJ20080321 18426390 PMC2474560

[B7] Bagchi S. Fredriksson R. Wallén-Mackenzie Å. (2015). *In Situ* Proximity Ligation Assay (PLA). Methods Mol. Biol. 1318, 149–159. 10.1007/978-1-4939-2742-5_15 26160573

[B8] Berardi M. J. Shih W. M. Harrison S. C. Chou J. J. (2011). Mitochondrial Uncoupling Protein 2 Structure Determined by NMR Molecular Fragment Searching. Nature 476 (7358), 109–113. 10.1038/nature10257 21785437 PMC3150631

[B9] Bernardi P. Rasola A. Forte M. Lippe G. (2015). The Mitochondrial Permeability Transition Pore: Channel Formation by F-ATP Synthase, Integration in Signal Transduction, and Role in Pathophysiology. Physiol. Rev. 95 (4), 1111–1155. 10.1152/physrev.00001.2015 26269524 PMC4600949

[B10] Bertholet A. M. Chouchani E. T. Kazak L. Angelin A. Fedorenko A. Long J. Z. (2019). H(+) Transport Is an Integral Function of the Mitochondrial ADP/ATP Carrier. Nature 571 (7766), 515–520. 10.1038/s41586-019-1400-3 31341297 PMC6662629

[B11] Bevilacqua L. Seifert E. L. Estey C. Gerrits M. F. Harper M.-E. (2010). Absence of Uncoupling Protein-3 Leads to Greater Activation of an Adenine Nucleotide Translocase-Mediated Proton Conductance in Skeletal Muscle Mitochondria from Calorie Restricted Mice. Biochimica Biophysica Acta (BBA) - Bioenergetics 1797 (8), 1389–1397. 10.1016/j.bbabio.2010.02.018 20206124

[B12] Boss O. Samec S. Dulloo A. Seydoux J. Patrick Muzzin P. Giacobino J.-P. (1997). Tissue-dependent Upregulation of Rat Uncoupling Protein-2 Expression in Response to Fasting or Cold. FEBS Lett. 412 (1), 111–114. 10.1016/s0014-5793(97)00755-2 9257701

[B13] Bouillaud F. (2009). UCP2, Not a Physiologically Relevant Uncoupler but a Glucose Sparing Switch Impacting ROS Production and Glucose Sensing. Biochimica Biophysica Acta (BBA) - Bioenergetics 1787 (5), 377–383. 10.1016/j.bbabio.2009.01.003 19413946

[B14] Brand M. D. Affourtit C. Esteves T. C. Green K. Lambert A. J. Miwa S. (2004). Mitochondrial Superoxide: Production, Biological Effects, and Activation of Uncoupling Proteins. Free Radic. Biol. Med. 37 (6), 755–767. 10.1016/j.freeradbiomed.2004.05.034 15304252

[B15] Brand M. D. Pakay J. L. Ocloo A. Kokoszka J. Wallace D. C. Brookes P. S. (2005). The Basal Proton Conductance of Mitochondria Depends on Adenine Nucleotide Translocase Content. Biochem. J. 392 (Pt 2), 353–362. 10.1042/BJ20050890 16076285 PMC1316271

[B16] Cannon B. Shabalina I. G. Kramarova T. V. Petrovic N. Nedergaard J. (2006). Uncoupling Proteins: A Role in Protection against Reactive Oxygen Species--Or Not? Biochimica Biophysica Acta (BBA) - Bioenergetics 1757 (5-6), 449–458. 10.1016/j.bbabio.2006.05.016 16806053

[B17] Cesaro L. Salvi M. (2010). Mitochondrial Tyrosine Phosphoproteome: New Insights from an Up-To-Date Analysis. Biofactors 36 (6), 437–450. 10.1002/biof.123 21072759

[B18] Chang A. H. K. Sancheti H. Garcia J. Kaplowitz N. Cadenas E. Han D. (2014). Respiratory Substrates Regulate S-Nitrosylation of Mitochondrial Proteins through a Thiol-dependent Pathway. Chem. Res. Toxicol. 27 (5), 794–804. 10.1021/tx400462r 24716714 PMC4033640

[B19] Chorev D. S. Baker L. A. Wu D. Beilsten-Edmands V. Rouse S. L. Zeev-Ben-Mordehai T. (2018). Protein Assemblies Ejected Directly from Native Membranes Yield Complexes for Mass Spectrometry. Science 362 (6416), 829–834. 10.1126/science.aau0976 30442809 PMC6522346

[B20] Doerner A. Pauschinger M. Badorff A. Noutsias M. Giessen S. Schulze K. (1997). Tissue-specific Transcription Pattern of the Adenine Nucleotide Translocase Isoforms in Humans. FEBS Lett. 414 (2), 258–262. 10.1016/s0014-5793(97)01000-4 9315697

[B21] Dolce V. Scarcia P. Iacopetta D. Palmieri F. (2005). A Fourth ADP/ATP Carrier Isoform in Man: Identification, Bacterial Expression, Functional Characterization and Tissue Distribution. FEBS Lett. 579 (3), 633–637. 10.1016/j.febslet.2004.12.034 15670820

[B22] Dupont P.-Y. Stepien G. (2011). Computational Analysis of the Transcriptional Regulation of the Adenine Nucleotide Translocator Isoform 4 Gene and its Role in Spermatozoid Glycolytic Metabolism. Gene 487 (1), 38–45. 10.1016/j.gene.2011.07.024 21827840

[B23] Echtay K. S. Esteves T. C. Pakay J. L. Jekabsons M. B. Lambert A. J. Portero-Otin M. (2003). A Signalling Role for 4-Hydroxy-2-Nonenal in Regulation of Mitochondrial Uncoupling. EMBO J. 22 (16), 4103–4110. 10.1093/emboj/cdg412 12912909 PMC175801

[B24] Feng J. Zhu M. Schaub M. C. Gehrig P. Roschitzki B. Lucchinetti E. (2008). Phosphoproteome Analysis of Isoflurane-Protected Heart Mitochondria: Phosphorylation of Adenine Nucleotide Translocator-1 on Tyr194 Regulates Mitochondrial Function. Cardiovasc Res. 80 (1), 20–29. 10.1093/cvr/cvn161 18558627

[B25] Friederich M. Nordquist L. Olerud J. Johansson M. Hansell P. Palm F. (2009). Identification and Distribution of Uncoupling Protein Isoforms in the Normal and Diabetic Rat Kidney. Adv. Exp. Med. Biol. 645, 205–212. 10.1007/978-0-387-85998-9_32 19227473

[B26] Friederich-Persson M. Aslam S. Nordquist L. Welch W. J. Wilcox C. S. Palm F. (2012). Acute Knockdown of Uncoupling Protein-2 Increases Uncoupling via the Adenine Nucleotide Transporter and Decreases Oxidative Stress in Diabetic Kidneys. PLoS One 7 (7), e39635. 10.1371/journal.pone.0039635 22768304 PMC3388100

[B27] Golozoubova V. Hohtola E. Matthias A. Jacobsson A. Cannon B. Nedergaard J. (2001). Only UCP1 Can Mediate Adaptive Nonshivering Thermogenesis in the Cold. FASEB J. 15 (11), 2048–2050. 10.1096/fj.00-0536fje 11511509

[B28] Guo R. Gu J. Zong S. Wu M. Yang M. (2018). Structure and Mechanism of Mitochondrial Electron Transport Chain. Biomed. J. 41 (1), 9–20. 10.1016/j.bj.2017.12.001 29673555 PMC6138618

[B29] Halestrap A. P. Richardson A. P. (2015). The Mitochondrial Permeability Transition: a Current Perspective on its Identity and Role in Ischaemia/reperfusion Injury. J. Mol. Cell. Cardiol. 78, 129–141. 10.1016/j.yjmcc.2014.08.018 25179911

[B30] Halestrap A. P. (2009). What Is the Mitochondrial Permeability Transition Pore? J. Mol. Cell. Cardiol. 46 (6), 821–831. 10.1016/j.yjmcc.2009.02.021 19265700

[B31] Halestrap A. P. Woodfield K.-Y. Connern C. P. (1997). Oxidative Stress, Thiol Reagents, and Membrane Potential Modulate the Mitochondrial Permeability Transition by Affecting Nucleotide Binding to the Adenine Nucleotide Translocase. J. Biol. Chem. 272 (6), 3346–3354. 10.1074/jbc.272.6.3346 9013575

[B32] Haworth R. A. Hunter D. R. M. (2000). Control of the Mitochondrial Permeability Transition Pore by High-Affinity ADP Binding at the ADP/ATP Translocase in Permeabilized Mitochondria. J. Bioenerg. Biomembr. 32 (1), 91–96. 10.1023/a:1005568630151 11768766

[B33] Hoang T. Kuljanin M. Smith M. D. Jelokhani-Niaraki M. (2015). A Biophysical Study on Molecular Physiology of the Uncoupling Proteins of the Central Nervous System. Biosci. Rep. 35 (4), e00226. 10.1042/BSR20150130 26182433 PMC4613710

[B34] Hunter D. R. Haworth R. A. (1979). The Ca2+-Induced Membrane Transition in Mitochondria. I. The protective mechanisms. Archives Biochem. Biophysics 195 (2), 453–459. 10.1016/0003-9861(79)90371-0 383019

[B35] Jekabsons M. B. Echtay K. S. Arechaga I. Brand M. D. (2003). Molecular Properties of Purified Human Uncoupling Protein 2 Refolded from Bacterial Inclusion Bodies. J. Bioenerg. Biomembr. 35 (5), 409–418. 10.1023/a:1027335713635 14740889

[B36] Khailova L. S. Prikhodko E. A. Dedukhova V. I. Mokhova E. N. Popov V. N. Skulachev V. P. (2006). Participation of ATP/ADP Antiporter in Oleate- and Oleate Hydroperoxide-Induced Uncoupling Suppressed by GDP and Carboxyatractylate. Biochimica Biophysica Acta (BBA) - Bioenergetics 1757 (9-10), 1324–1329. 10.1016/j.bbabio.2006.04.024 16765906

[B37] Klingenberg M. Appel M. (1989). The Uncoupling Protein Dimer Can Form a Disulfide Cross-Link between the Mobile C-Terminal SH Groups. Eur. J. Biochem. 180 (1), 123–131. 10.1111/j.1432-1033.1989.tb14622.x 2495940

[B38] Klingenberg M. (2008). The ADP and ATP Transport in Mitochondria and its Carrier. Biochimica Biophysica Acta (BBA) - Biomembr. 1778 (10), 1978–2021. 10.1016/j.bbamem.2008.04.011 18510943

[B39] Komelina N. P. Amerkhanov Z. G. (2010). A Comparative Study of the Inhibitory Effects of Purine Nucleotides and Carboxyatractylate on the Uncoupling Protein-3 and Adenine Nucleotide Translocase. Acta Biochim. Pol. 57 (4), 413–419. 10.18388/abp.2010_2427 21152446

[B40] Liu Y. Chen X. J. (2013). Adenine Nucleotide Translocase, Mitochondrial Stress, and Degenerative Cell Death. Oxidative Med. Cell. Longev. 2013, 1–10. 10.1155/2013/146860 PMC3732615 23970947

[B41] Long J. Z. Svensson K. J. Bateman L. A. Lin H. Kamenecka T. Lokurkar I. A. (2016). The Secreted Enzyme PM20D1 Regulates Lipidated Amino Acid Uncouplers of Mitochondria. Cell. 166 (2), 424–435. 10.1016/j.cell.2016.05.071 27374330 PMC4947008

[B42] Lu Y.-W. Acoba M. G. Selvaraju K. Huang T.-C. Nirujogi R. S. Sathe G. (2017). Human Adenine Nucleotide Translocases Physically and Functionally Interact with Respirasomes. MBoC 28 (11), 1489–1506. 10.1091/mbc.E17-03-0195 28404750 PMC5449148

[B43] Mailloux R. J. Harper M.-E. (2011). Uncoupling Proteins and the Control of Mitochondrial Reactive Oxygen Species Production. Free Radic. Biol. Med. 51 (6), 1106–1115. 10.1016/j.freeradbiomed.2011.06.022 21762777

[B44] Malingriaux E. A. Rupprecht A. Gille L. Jovanovic O. Jezek P. Jaburek M. (2013). Fatty Acids Are Key in 4-Hydroxy-2-Nonenal-Mediated Activation of Uncoupling Proteins 1 and 2. PLoS One 8 (10), e77786. 10.1371/journal.pone.0077786 24204965 PMC3810126

[B45] Matthias A. Ohlson K. B. E. Fredriksson J. M. Jacobsson A. Nedergaard J. Cannon B. (2000). Thermogenic Responses in Brown Fat Cells Are Fully UCP1-dependent. UCP2 or UCP3 do not substitute for UCP1 in adrenergically or fatty scid-induced thermogenesis. J. Biol. Chem. 275 (33), 25073–25081. 10.1074/jbc.M000547200 10825155

[B46] Mielke C. Lefort N. McLean C. G. Cordova J. M. Langlais P. R. Bordner A. J. (2014). Adenine Nucleotide Translocase Is Acetylated *In Vivo* in Human Muscle: Modeling Predicts a Decreased ADP Affinity and Altered Control of Oxidative Phosphorylation. Biochemistry 53 (23), 3817–3829. 10.1021/bi401651e 24884163 PMC4067143

[B47] Mills E. M. Banks M. L. Sprague J. E. Finkel T. (2003). Pharmacology: Uncoupling the Agony from Ecstasy. Nature 426 (6965), 403–404. 10.1038/426403a 14647371

[B48] Moreno-Lastres D. Fontanesi F. García-Consuegra I. Martín M. A. Arenas J. Barrientos A. (2012). Mitochondrial Complex I Plays an Essential Role in Human Respirasome Assembly. Cell. Metab. 15 (3), 324–335. 10.1016/j.cmet.2012.01.015 22342700 PMC3318979

[B49] Nicholls D. G. Bernson V. S. M. Heaton G. M. (1978). The Identification of the Component in the Inner Membrane of Brown Adipose Tissue Mitochondria Responsible for Regulating Energy Dissipation. Exp. Suppl. 32, 89–93. 10.1007/978-3-0348-5559-4_9 348493

[B50] Nicholls D. G. (2021). Mitochondrial Proton Leaks and Uncoupling Proteins. Biochimica Biophysica Acta (BBA) - Bioenergetics 1862 (7), 148428. 10.1016/j.bbabio.2021.148428 33798544

[B51] Parker N. Affourtit C. Vidal-Puig A. Brand M. D. (2008). Energization-dependent Endogenous Activation of Proton Conductance in Skeletal Muscle Mitochondria. Biochem. J. 412 (1), 131–139. 10.1042/BJ20080006 18251717 PMC2474556

[B52] Pebay-Peyroula E. Dahout-Gonzalez C. Kahn R. Trézéguet V. Lauquin G. J.-M. Brandolin G. (2003). Structure of Mitochondrial ADP/ATP Carrier in Complex with Carboxyatractyloside. Nature 426 (6962), 39–44. 10.1038/nature02056 14603310

[B53] Pecqueur C. Bui T. Gelly C. Hauchard J. Barbot C. Bouillaud F. (2008). Uncoupling Protein‐2 Controls Proliferation by Promoting Fatty Acid Oxidation and Limiting Glycolysis‐derived Pyruvate Utilization. FASEB J. 22 (1), 9–18. 10.1096/fj.07-8945com 17855623

[B54] Queiroga C. S. F. Almeida A. S. Martel C. Brenner C. Alves P. M. Vieira H. L. A. (2010). Glutathionylation of Adenine Nucleotide Translocase Induced by Carbon Monoxide Prevents Mitochondrial Membrane Permeabilization and Apoptosis. J. Biol. Chem. 285 (22), 17077–17088. 10.1074/jbc.M109.065052 20348099 PMC2878049

[B55] Schiffer T. A. Gustafsson H. Palm F. (2018). Kidney Outer Medulla Mitochondria Are More Efficient Compared with Cortex Mitochondria as a Strategy to Sustain ATP Production in a Suboptimal Environment. Am. J. Physiology-Renal Physiology 315 (3), F677–F681. 10.1152/ajprenal.00207.2018 29846107

[B56] Söderberg O. Gullberg M. Jarvius M. Ridderstråle K. Leuchowius K.-J. Jarvius J. (2006). Direct Observation of Individual Endogenous Protein Complexes *In Situ* by Proximity Ligation. Nat. Methods 3 (12), 995–1000. 10.1038/nmeth947 17072308

[B57] Stepien G. Torroni A. Chung A. B. Hodge J. A. Wallace D. C. (1992). Differential Expression of Adenine Nucleotide Translocator Isoforms in Mammalian Tissues and during Muscle Cell Differentiation. J. Biol. Chem. 267 (21), 14592–14597. 10.1016/s0021-9258(18)42082-0 1378836

[B58] Toyomizu M. Ueda M. Sato S. Seki Y. Sato K. Akiba Y. (2002). Cold-induced Mitochondrial Uncoupling and Expression of Chicken UCP and ANT mRNA in Chicken Skeletal Muscle. FEBS Lett. 529 (2-3), 313–318. 10.1016/s0014-5793(02)03395-1 12372620

[B59] Vidal-Puig A. J. Grujic D. Zhang C.-Y. Hagen T. Boss O. Ido Y. (2000). Energy Metabolism in Uncoupling Protein 3 Gene Knockout Mice. J. Biol. Chem. 275 (21), 16258–16266. 10.1074/jbc.M910179199 10748196

[B60] Vozza A. Parisi G. De Leonardis F. Lasorsa F. M. Castegna A. Amorese D. (2014). UCP2 Transports C4 Metabolites Out of Mitochondria, Regulating Glucose and Glutamine Oxidation. Proc. Natl. Acad. Sci. U.S.A. 111 (3), 960–965. 10.1073/pnas.1317400111 24395786 PMC3903233

[B61] Yamamoto A. Hasui K. Matsuo H. Okuda K. Abe M. Matsumoto K. (2015). Bongkrekic Acid Analogue, Lacking One of the Carboxylic Groups of its Parent Compound, Shows Moderate but pH-Insensitive Inhibitory Effects on the Mitochondrial ADP/ATP Carrier. Chem. Biol. Drug Des. 86 (5), 1304–1322. 10.1111/cbdd.12594 26032198

[B62] Yan L.-J. Sohal R. S. (1998). Mitochondrial Adenine Nucleotide Translocase Is Modified Oxidatively during Aging. Proc. Natl. Acad. Sci. U.S.A. 95 (22), 12896–12901. 10.1073/pnas.95.22.12896 9789011 PMC23645

[B63] Zhao X. León I. R. Bak S. Mogensen M. Wrzesinski K. Højlund K. (2011). Phosphoproteome Analysis of Functional Mitochondria Isolated from Resting Human Muscle Reveals Extensive Phosphorylation of Inner Membrane Protein Complexes and Enzymes. Mol. Cell. Proteomics 10 (1), M110. 10.1074/mcp.M110.000299 PMC3013442 20833797

[B64] Zhou H.-X. Cross T. A. (2013). Influences of Membrane Mimetic Environments on Membrane Protein Structures. Annu. Rev. Biophys. 42, 361–392. 10.1146/annurev-biophys-083012-130326 23451886 PMC3731949

[B65] Zoonens M. Comer J. Masscheleyn S. Pebay-Peyroula E. Chipot C. Miroux B. (2013). Dangerous Liaisons between Detergents and Membrane Proteins. The Case of Mitochondrial Uncoupling Protein 2. J. Am. Chem. Soc. 135 (40), 15174–15182. 10.1021/ja407424v 24021091

