# Supplementary figures and images for: Mitochondrial Respiration-Dependent ANT2-UCP2 Interaction
Source: Front Physiol. 2022 May 25;13:866590. doi: 10.3389/fphys.2022.866590 (PMC9177158; doi:10.3389/fphys.2022.866590)

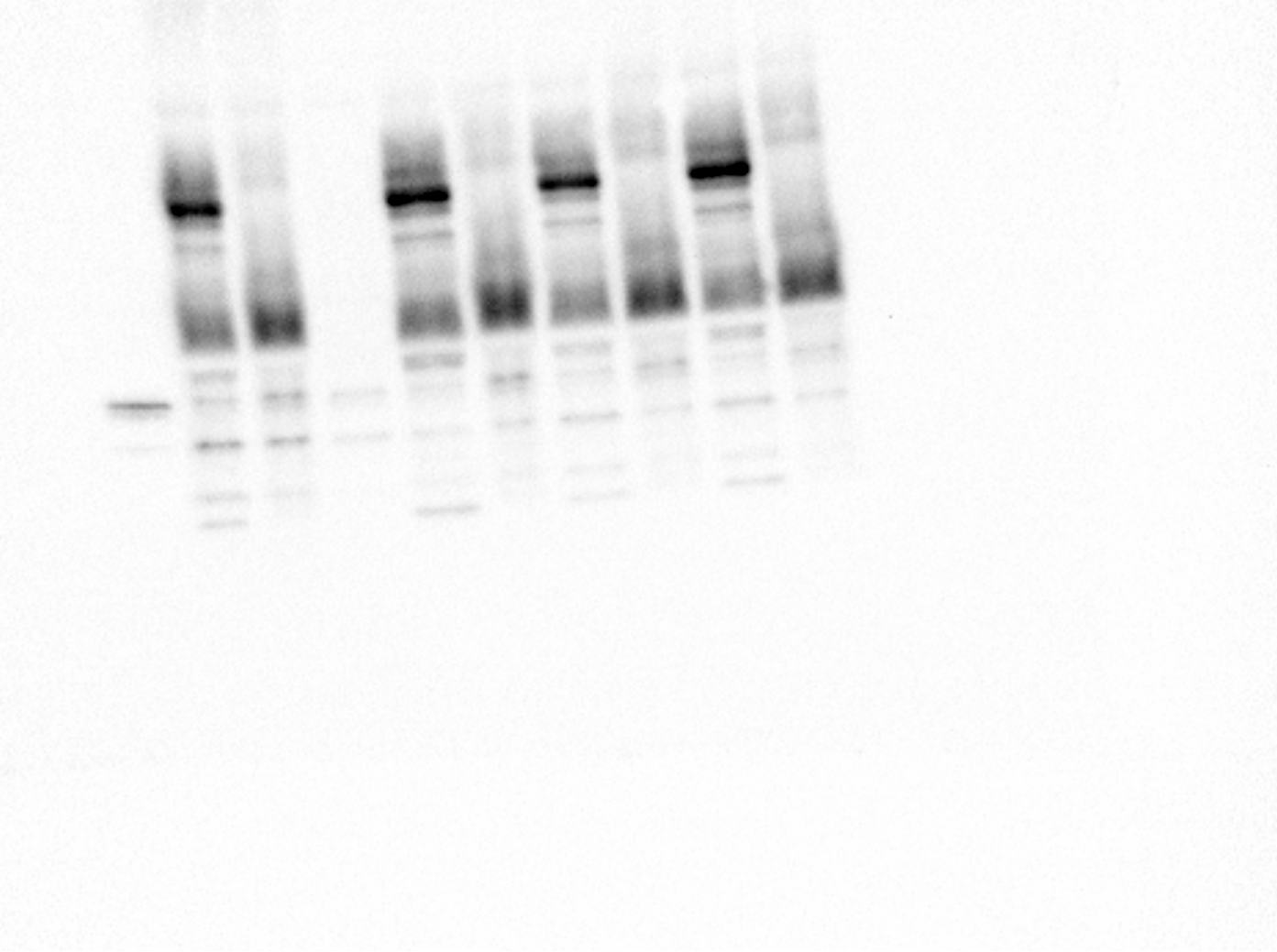

Supplement: Supplementary file 1 [file DataSheet1.ZIP › WB blots from Frontiers/190828 UCP2 3sec original.tif]

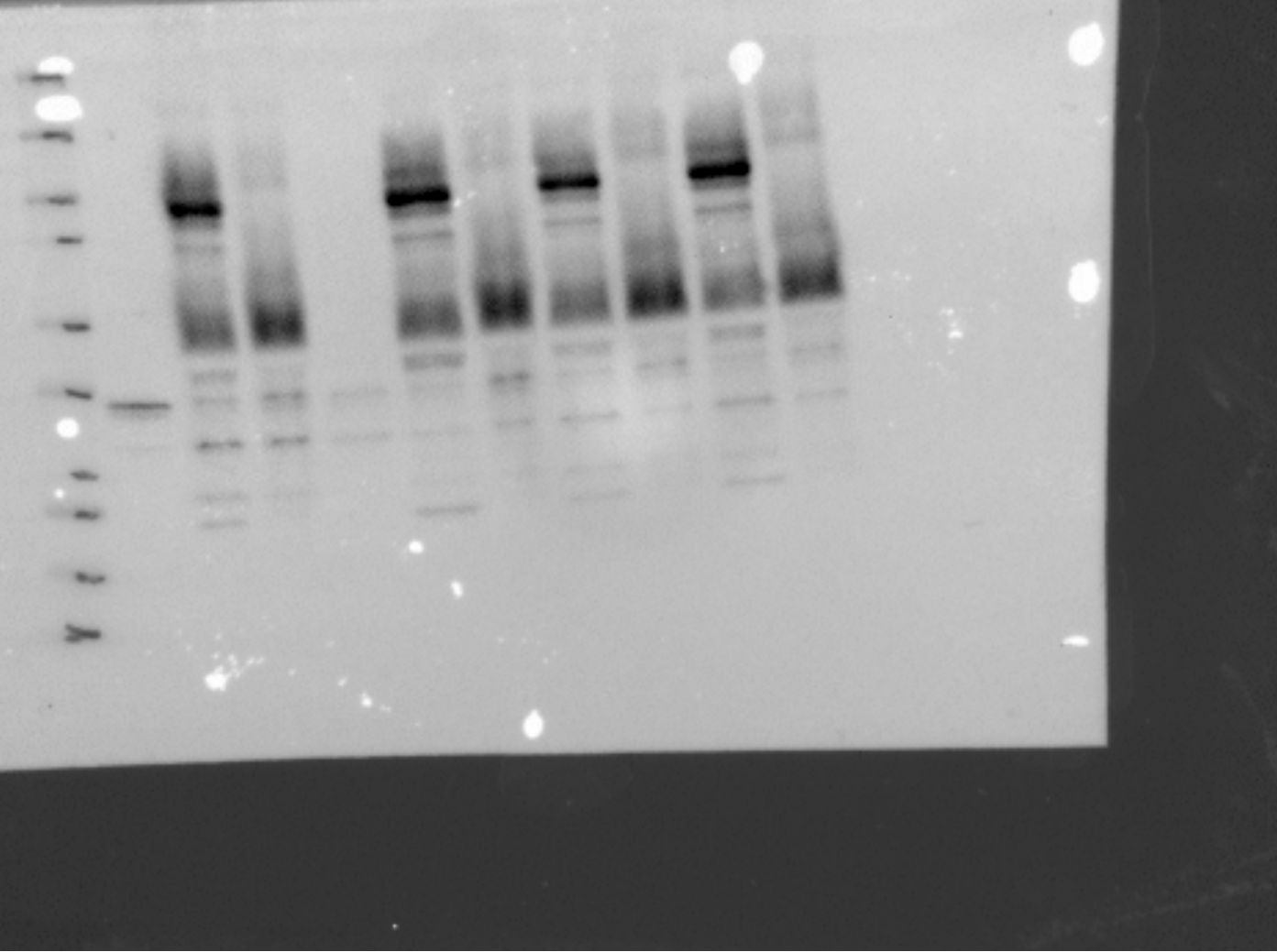

Supplement: Supplementary file 1 [file DataSheet1.ZIP › WB blots from Frontiers/190828 UCP2 merged ladder.tif]

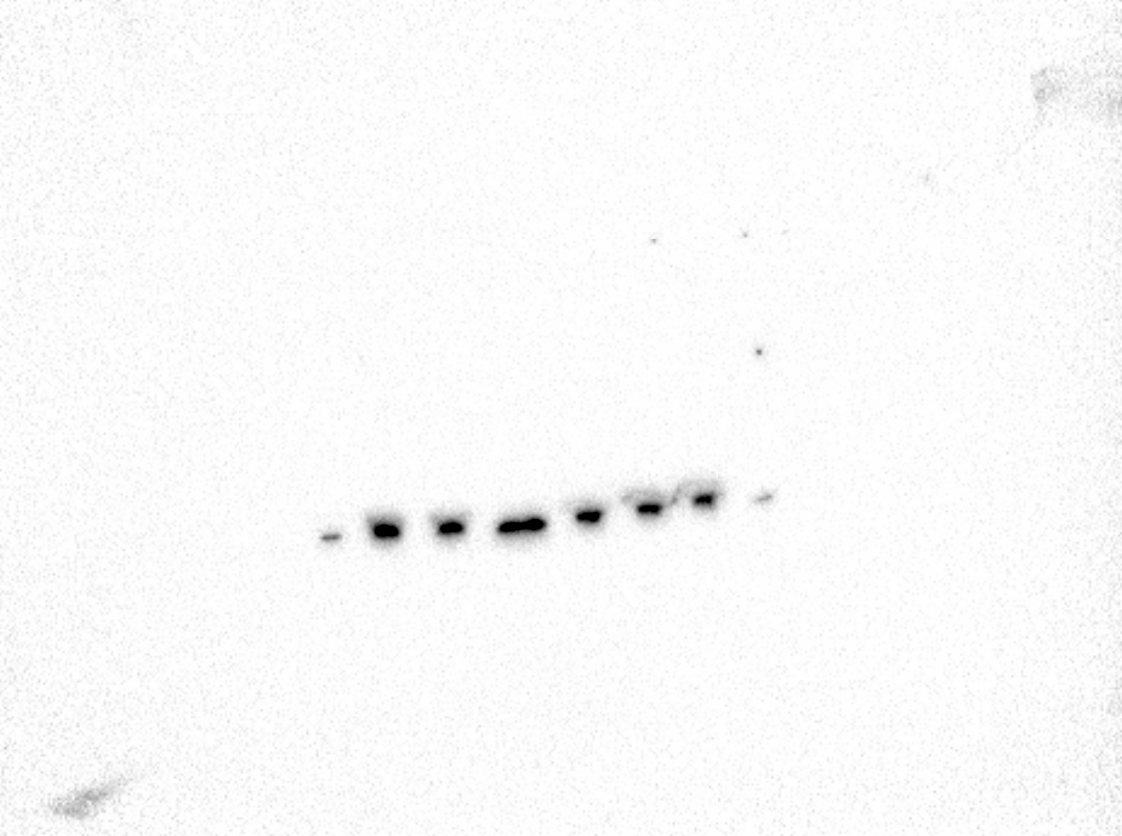

Supplement: Supplementary file 1 [file DataSheet1.ZIP › WB blots from Frontiers/200213 Vinculin for UCP2.tif]

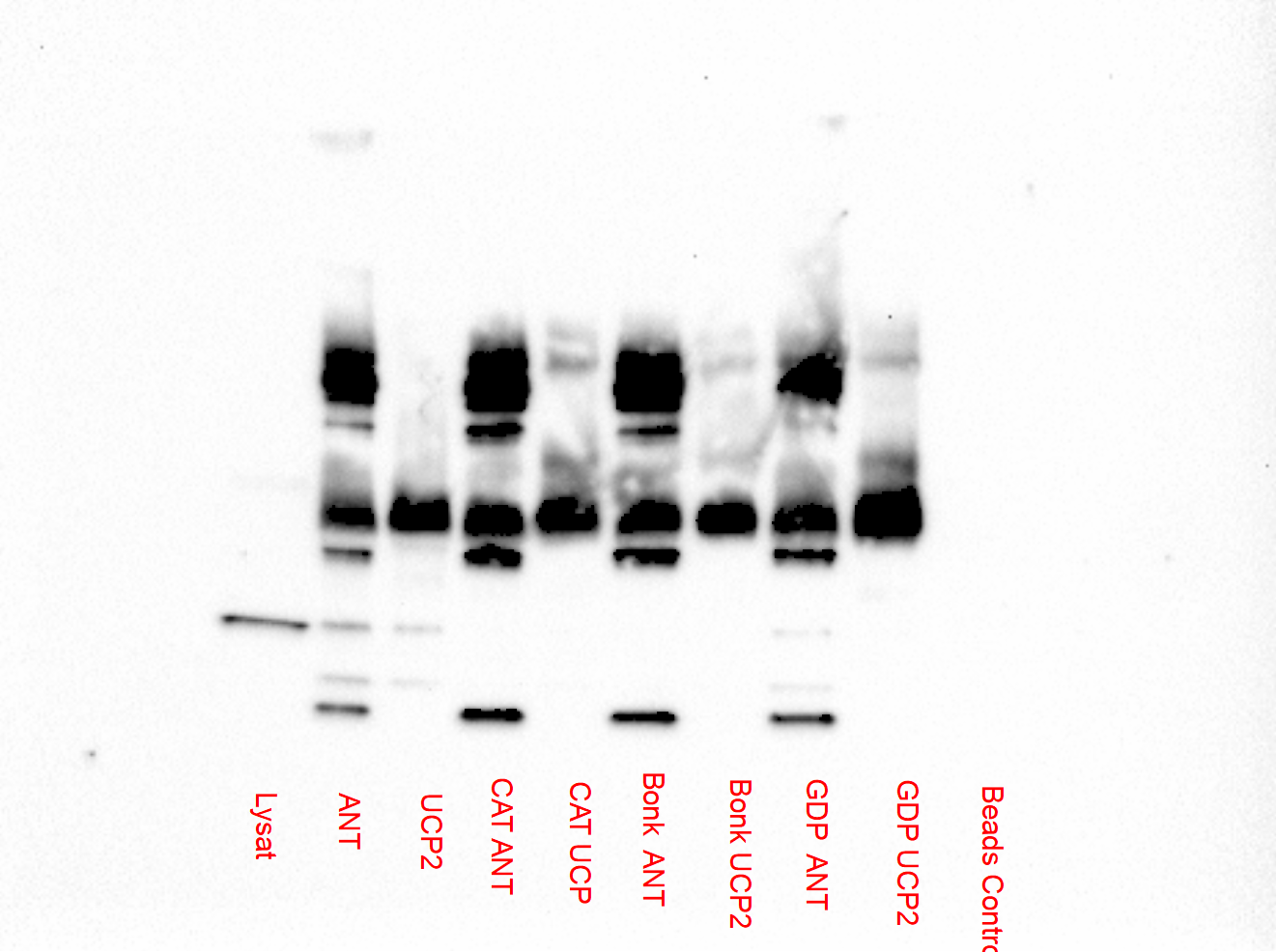

Supplement: Supplementary file 1 [file DataSheet1.ZIP › WB blots from Frontiers/ANT 190206 37sek.tif]

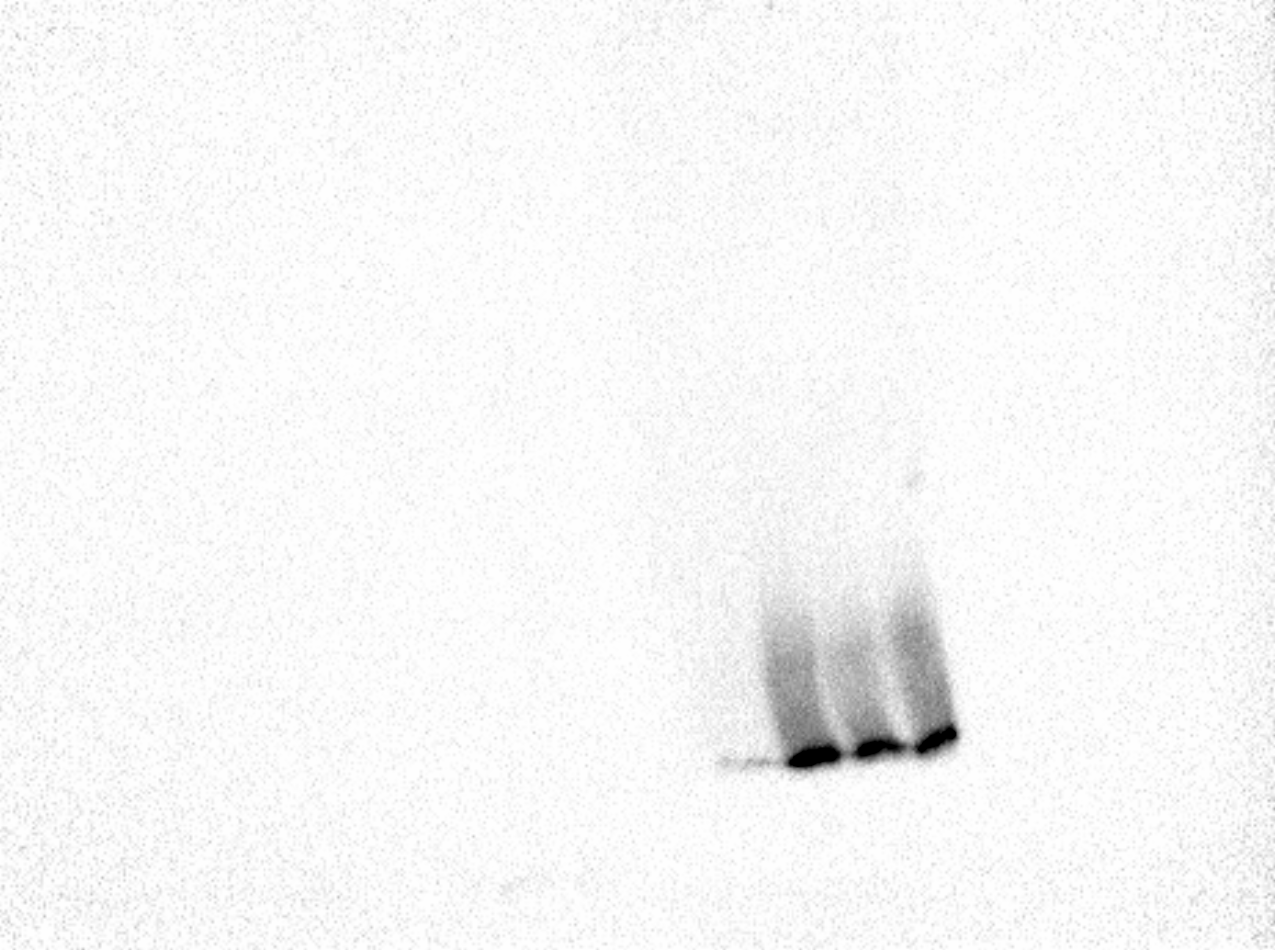

Supplement: Supplementary file 1 [file DataSheet1.ZIP › WB blots from Frontiers/ANT 2 2020-02-12 11hr 36min_Exposure_27.0sec.tif]

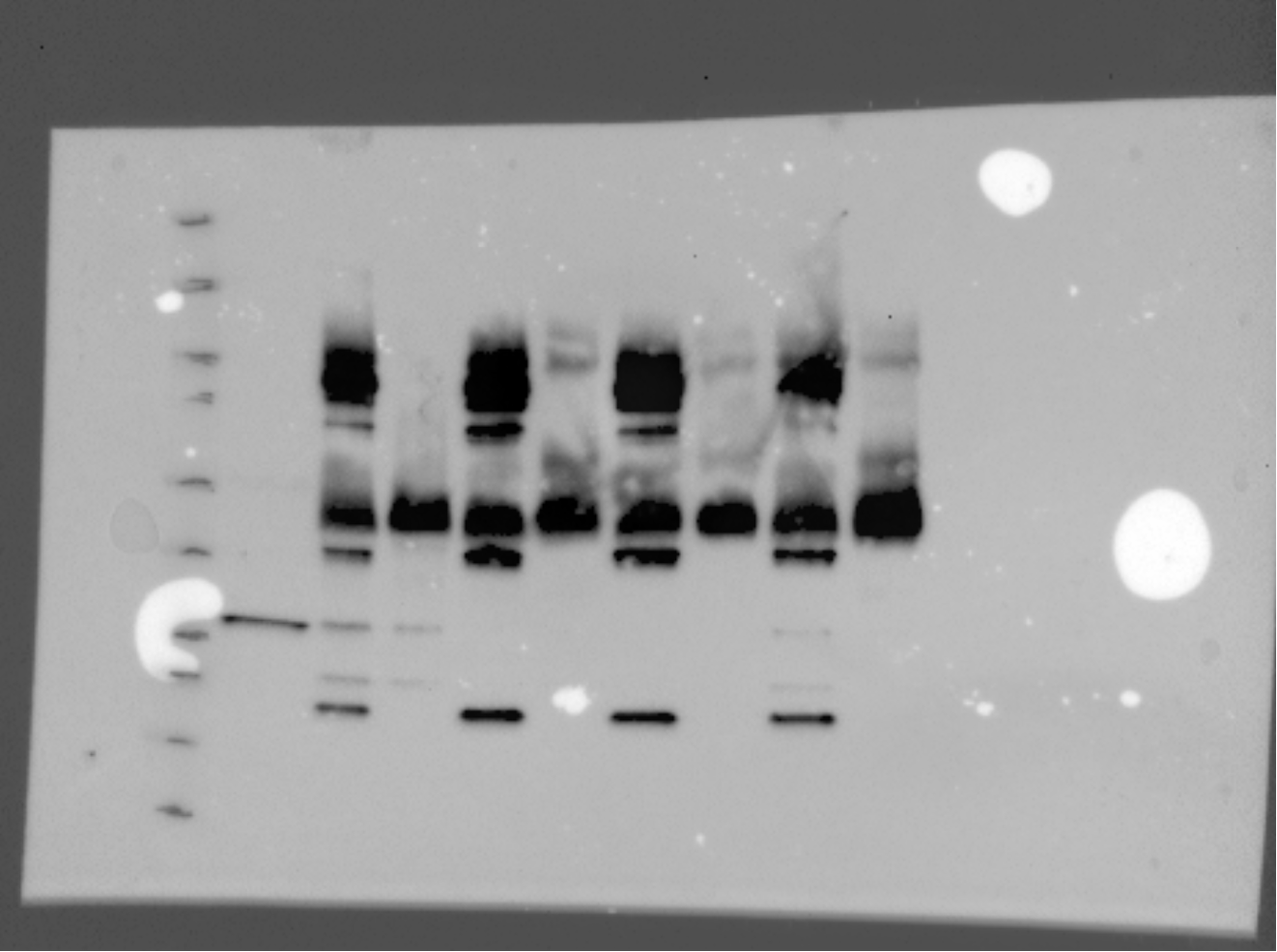

Supplement: Supplementary file 1 [file DataSheet1.ZIP › WB blots from Frontiers/ANT merged ladder 190206.tif]

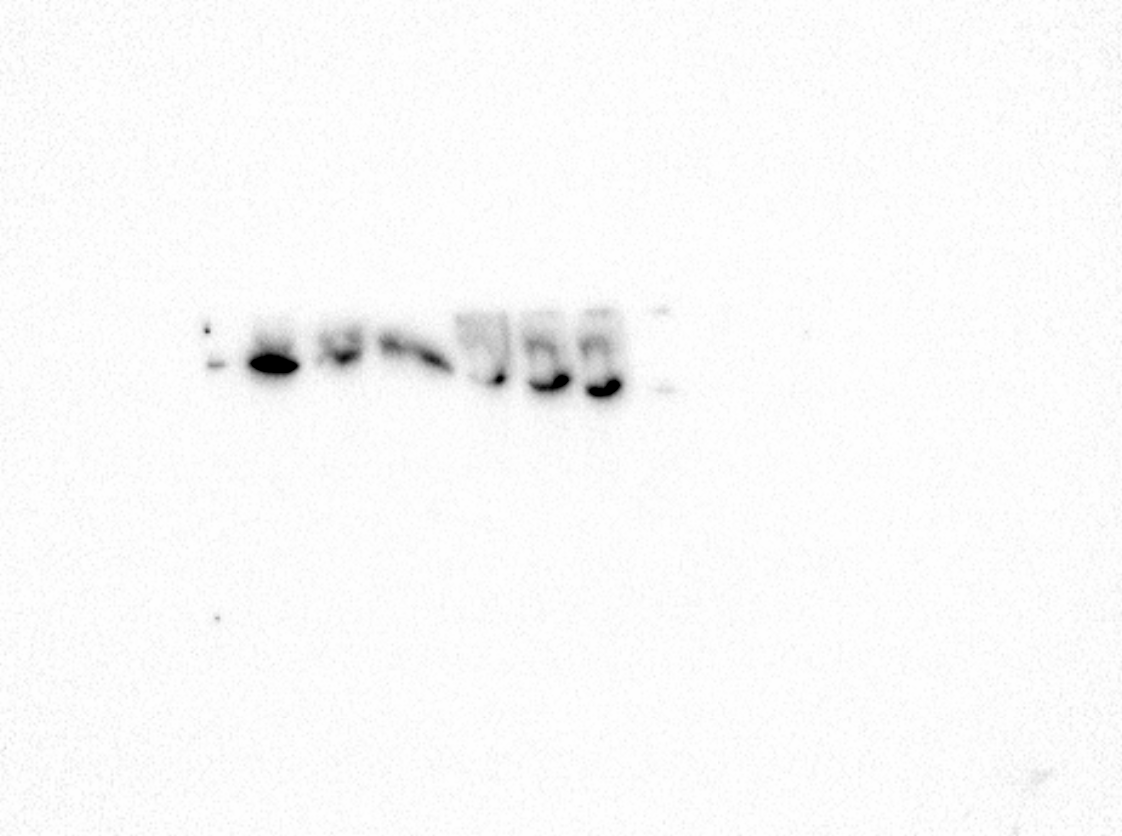

Supplement: Supplementary file 1 [file DataSheet1.ZIP › WB blots from Frontiers/UCP2 efter KD celler 2020-02-13 11hr 30min_Exposure_5.0sec.tif]

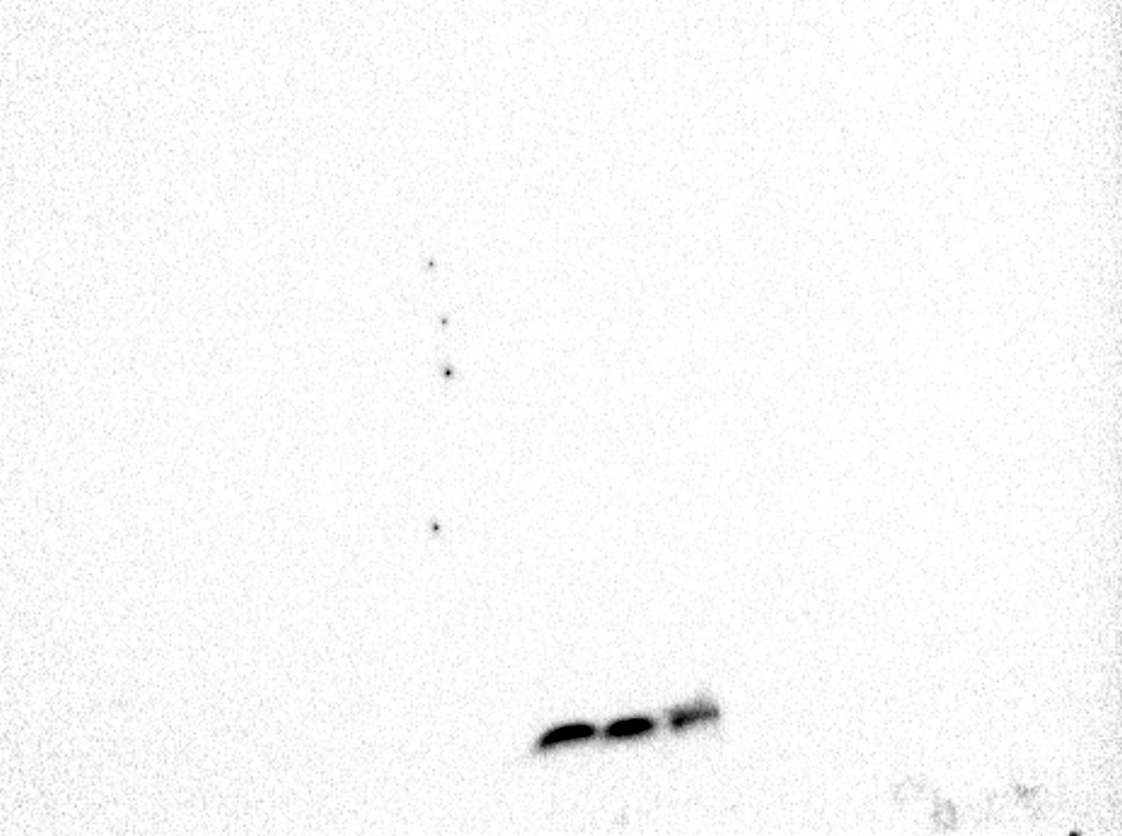

Supplement: Supplementary file 1 [file DataSheet1.ZIP › WB blots from Frontiers/vinculin för ANT2 2020-02-13 11hr 27min_Exposure_47.0sec.tif]
